# Risk factors for posttraumatic empyema in diaphragmatic injuries

**DOI:** 10.1186/s13017-022-00453-9

**Published:** 2022-09-13

**Authors:** Alberto Federico García, Fernando Rodríguez, Álvaro Sánchez, Isabella Caicedo-Holguín, Carlos Gallego-Navarro, María Paula Naranjo, Yaset Caicedo, Daniela Burbano, Diana Felisa Currea-Perdomo, Carlos A. Ordoñez, Juan Carlos Puyana

**Affiliations:** 1 Division of Trauma and Acute Care Surgery, Department of Surgery, Department of Intensive Care, Fundación Valle del Lili, Cra 98 No. 18–49, 760032 Cali, Colombia; 2 Division of Trauma and Acute Care Surgery, Department of Surgery, Universidad del Valle, Cali, Colombia; 3 Department of General Surgery, Universidad Icesi, Cali, Colombia; 4 Division of Thoracic Surgery, Department of Surgery, Fundación Valle del Lili, Cra 98 No. 18–49, 760032 Cali, Colombia; 5 Centro de Investigaciones Clínicas (CIC), Fundación Valle del Lili, Cra 98 No. 18–49, 760032 Cali, Colombia; 6 Department of Cardiovascular Surgery, Mayo Clinic, Rochester, MN USA; 7 Department of General Surgery, Universidad de Sanitas, Bogotá, Colombia; 8 Department of General Surgery, Universidad de Caldas, Manizales, Colombia; 9 Division of Oncologic Surgery, Fundación Valle del Lili, Cra No. 18–49, 760032 Cali, Colombia; 10 Professor of Surgery Director Global Health, Critical Care and Clinical Translational Surgery, University of Pittsburgh, Pittsburgh, PA USA

**Keywords:** Empyema, Diaphragmatic injury, Thoracic injuries, Wounds, Penetrating

## Abstract

**Background:**

Penetrating diaphragmatic injuries are associated with a high incidence of posttraumatic empyema. We analyzed the contribution of trauma severity, specific organ injury, contamination severity, and surgical management to the risk of posttraumatic empyema in patients who underwent surgical repair of diaphragmatic injuries at a level 1 trauma center.

**Methods:**

This is a retrospective review of the patients who survived more than 48 h. Univariate OR calculations were performed to identify potential risk factors. Multiple logistic regression was used to calculate adjusted ORs and identify independent risk factors.

**Results:**

We included 192 patients treated from 2011 to 2020. There were 169 (88.0) males. The mean interquartile range, (IQR) of age, was 27 (22–35) years. Gunshot injuries occurred in 155 subjects (80.7%). Mean (IQR) NISS and ATI were 29 (18–44) and 17 (10–27), respectively. Thoracic AIS was > 3 in 38 patients (19.8%). Hollow viscus was injured in 105 cases (54.7%): stomach in 65 (33.9%), colon in 52 (27.1%), small bowel in 42 (21.9%), and duodenum in 10 (5.2%). Visible contamination was found in 76 patients (39.6%). Potential thoracic contamination was managed with a chest tube in 128 cases (66.7%), with transdiaphragmatic pleural lavage in 42 (21.9%), and with video-assisted thoracoscopy surgery or thoracotomy in 22 (11.5%). Empyema occurred in 11 patients (5.7%). Multiple logistic regression identified thoracic AIS > 3 (OR 6.4, 95% CI 1.77–23. 43), and visible contamination (OR 5.13, 95% IC 1.26–20.90) as independent risk factors. The individual organ injured, or the method used to manage the thoracic contamination did not affect the risk of posttraumatic empyema.

**Conclusion:**

The severity of the thoracic injury and the presence of visible abdominal contamination were identified as independent risk factors for empyema after penetrating diaphragmatic trauma.

## Background

The presence of an abnormal communication between the abdominal and thoracic cavities due to a diaphragmatic injury is a potential source of chest infection. This condition is present in most trauma patients with penetrating diaphragmatic injury. However, few studies have explored the factors that might contribute to posttraumatic empyema in the context of diaphragmatic injury [1, 2]. This type of trauma can be caused by both penetrating thoracoabdominal injuries and severe blunt trauma [3, 4]. Diaphragmatic injury in trauma patients is rare [2], except for patients with thoracoabdominal penetrating trauma were the incidence ranges between 30 and 40% [5, 6].

Usually, diaphragmatic injuries are complex and often associated with other organ injuries [2, 7, 8]. A posttraumatic empyema may result from either a direct contamination of the pleural cavity associated with skin disruption (in chest tube placement or gunshot wound) or by a diaphragmatic wall disruption by direct, hematogenous, or lymphatic spread of abdominal or thoracic contamination. [9, 10]. The proportion of posttraumatic empyema in patients with chest trauma has been reported between 1.6 and 25% [11–15]. In contrast, thoracoabdominal trauma and diaphragmatic injury case series had reported a low rate ranging between 1.4 and 1.8% [1, 2]. Several interventions have been proposed to reduce the risk of infection in the context of pleural contamination secondary to hollow viscus perforation, such as transdiaphragmatic pleural lavage (TDPL), chest tube placement, and thoracic lavage by thoracoscopy or thoracotomy. Nevertheless, it is unclear whether these interventions are beneficial for all patients with diaphragmatic traumatic injury. This study aims to explore which factors related to the physiological status, injury severity, and surgical techniques contribute to the development of posttraumatic empyema in patients with diaphragmatic injury.

## Material and methods

### Study design

We performed a retrospective study of the patients treated at two university hospitals, equivalent to a level-1 trauma center in a city in the country of Colombia. This study was approved for conduction and publication by institutional review board and ethics committees of both institutions. No funds were received for the planning, conduction, analysis, and presentation of the results of the study.

### Settings and participants

We included patients 15 years or older with a diaphragmatic injury, treated with laparotomy or laparoscopy from 2008 to 2020. The exclusion criteria were thoracotomy for any indication different to managing the contamination, transfer to another facility during the first 5 days following the index surgery or death within the first 48 h.

### Variables and data collection

In order to identify the patients, the medical records of all patients with ICD-10 diagnosis of trauma (S200-S399) who underwent exploratory laparotomy or laparoscopy, pulmonary decortication either thoracoscopic or by thoracotomy and suture of diaphragmatic laceration were reviewed. The patients who met the criteria were included in the analysis.

Data analysis included variables such as demographics, trauma mechanism, hemodynamic parameters, trauma severity scores, associated injuries, initial surgical intervention, severity of the abdominal contamination, and procedures used to control the thoracic contamination. The diaphragmatic injury was described according to AAST-OIS classification, the AIS classification to describe the injuries of the thoracic organs and the injuries of the abdominal organs as present or absent [16]. The AIS classification was scored by the investigators during the chart revision process. Pulmonary empyema was defined as the surgical finding of pus or abscess in the pleural cavity described by a surgeon or the identification of bacteria in a thoracic cavity specimen surgically or percutaneously drained. Patients were divided into two groups, the ones who developed empyema during their hospital stay and the ones who did not.

Abdominal contamination was defined as absent when hollow viscus injury was not present. Non-visible, in cases where hollow viscus injury was present but no spillage of intestinal contents occurred. And visible when spillage was identified, and extended to an abdominal quadrant or also when more than one quadrant was compromised. Severe thoracic trauma was defined when the patient had chest AIS grade > 3.

#### Surgical techniques

The procedures used to manage the pleural contamination were: chest tube alone, pleural irrigation at the time of the chest tube placement, TDPL, and a pleural cavity lavage by thoracoscopy or thoracotomy. Each procedure was used at the discretion of the treating surgeon.

### Statistical analysis

Statistical analysis was performed using STATA 15.1 (College Station, TX). Continuous variables were described as median and interquartile range (IQR), and categorical variables as absolute and relative frequencies. Categorical variables were compared with exact Fisher’s test: Continuous variables with Wilcoxon–Mann–Whitney test. We explored the potential factors associated with the risk of posttraumatic empyema by univariate logistical regressions. Variables with a statistical significance of < 0.2 were considered relevant for the multivariate analysis. Given the limitation by the number of outcomes, separate analyses were performed to identify variables to the patient's reserve, the severity of the trauma, and the inoculum magnitude. A final model was constructed with the selected variables. The possible contribution of the procedures used to manage the pleural contamination to empyema was evaluated with logistic regression, stratifying the procedures according to their apparent risk. The result was adjusted by the variables identified in the previous risk model. Adjusted odds ratio and its 95% confidence interval were reported. The model's discriminative ability was evaluated by receiver operating curve (ROC) calculating its area under the curve (AUC) and the goodness to fit with the Hosmer–Lemeshow test. All statistical tests were 2-tailed, and a *P *value < 0.05 was considered significant.

## Results

There were 233 patients included; the median (IQR) age was 27 (22–35). Posttraumatic empyema was identified in 19 (8.2%) patients of all the patients who met the inclusion criteria. Table 1 shows demographic characteristics and the variables at arrival characterized by two groups: patients with empyema and patients without empyema. The most common mechanism of injury was gunshot wound in 170 of the cases. Nearly 10% of these patients presented posttraumatic empyema. At arrival to the emergency department, most of the patients had median a RTS score of 7.84 median (IQR) (7.11–7.84). Median (IQR) of ISS was 25 (14–34), similar in both groups as seen in Table 1. The NISS and ATI were higher in the empyema group.

**Table 1:** Risk factors for empyema after diaphragmatic trauma. General information

Variable	Value	Empyema		*p*
		No	Yes	
Patients, *n* (%)	233	214 (91.8%)	19 (8.2%)	
Age, median (IQR)	27 (22–35)	27 (22–35)	26 (22–30)	0.405^1^
Sex				0.594^2^
Female, *n* (%)	28 (12.0)	26 (12.5)	2 (10.5)	
Male, *n* (%)	205 (88.0)	188 (87.9)	17 (89.5)	
Trauma mechanism				0.365^2^
MVA, *n* (%)	5 (2.2)	4 (1.9)	1 (5.3)	
Assault, *n* (%)	3 (1.3)	3 (1.4)	0 (–)	
Explosion, *nn* (%)	4 (1.7)	4 (1.9)	0 (–)	
Stab wound, *n* (%)	49 (21.0)	48 (22.4)	1 (5.3)	
Gunshot, *n* (%)	170 (72.9)	153 (71.5)	17 (89.4)	
Other, blunt, *n* (%)	2 (0.9)	2 (0.9)	0 (–)	
SBP, mm Hg, median (IQR)	112 (97–130)	112.5 (97–130)	110 (86–127)	0.426^1^
RR, breath/min, median (IQR)	22 (19–26)	22 (19–26)	20 (18–26)	0.110^1^
GCS, median (IQR)	15 (15–15)	15 (15–15)	15 (14–15)	0.423^1^
RTS, median (IQR)	7.84 (7.11–7.84)	7.84 (7.11–7.84)	7.84 (7.11–7.84)	0.672^1^
ISS, median (IQR)	25 (14–34)	23 (14–33)	29 (14–35)	0.241^1^
NISS, median (IQR)	29 (18–43)	29 (18–43)	41 (34–50)	0.001^1^
ATI, median (IQR)	17 (10–26)	16 (9–26)	23 (13–38)	0.031^1^
Associated trauma				
Head/neck, *n* (%)	23 (9.9)	21 (9.8)	2 (10.5)	1.000^2^
Face, *n* (%)	42 (18.0)	35 (16.4)	7 (36.8)	0.054^2^
Extremities, *n* (%)	64 (27.5)	58 (27.1)	6 (31.6)	0.789^2^
External, *n* (%)	95 (40.8)	87 (40.7)	8 (42.1)	1.000^2^

*IQR* interquartile rank, *MVA* motor vehicle accident, *SBP* systolic blood pressure, *RR* respiratory rate, *RTS* Revised Trauma Score, *ISS* Injury Severity Score, *NISS* New Injury Severity Score, *ATI* Abdominal Trauma Index

^1^Wilkoxon–Mann–Whitney

^2^Fisher’s exact test

Table 2 depicts trauma severity variables in patients with and without posttraumatic empyema. In Table 2, the thoracic AIS seems to be related to posttraumatic empyema. In 18.6% of the patients with thoracic AIS > 3 presented empyema, meanwhile in 5.7% of the patients with thoracic AIS ≤ 3 empyema was present. Empyema occurred in 11.4% of the patients with hollow viscus injuries. The stomach was the most frequently abdominal injured organ. The left diaphragm was the most frequently injured. Regarding cases with abdominal contamination, hollow viscus perforation with or without visible contamination represented 73.3% of the cases of empyema. Interestingly, 5 patients with no hollow viscus injury developed empyema.

**Table 2:** Risk factors for empyema after diaphragmatic trauma. Trauma description

Variable	Total	Empyema		*p*
		No	Yes	
Thoracic AIS				0.11^1^
≤ 3, *n* (%)	190 (81.6)	179 (83.6)	11 (57.9)	
> 3, *n* (%)	43 (18.4)	35 (16.4)	8 (42.1)	
Abdominal AIS				0.670^2^
≤ 3, *n* (%)	124 (53.2)	113 (52.8)	11 (57.9)	
> 3, *n* (%)	109 (46.8)	101 (47.2)	8 (42.1)	
ATI				0.199^1^
< 15, *n* (%)	104 (44.6)	99 (46.3)	5 (26.3)	
15–24, *n* (%)	61 (26.2)	55 (25.7)	6 (31.6)	
≥ 25, *n* (%)	68 (29.2)	60 (28.0)	8 (42.1)	
Organ injured				
Any hollow viscus, *n* (%)	122 (52.4)	108 (50.5)	14 (73.7)	0.052^2^
Stomach, *n* (%)	76 (32.6)	66 (30.6)	10 (52.6)	0.052^2^
Duodenum, *n* (%)	11 (4.7)	10 (4.7)	1 (5.2)	1.000^1^
Small bowel, *n* (%)	46 (19.4)	41 (19.1)	5 (26.3)	0.546^1^
Colon, *n* (%)	62 (26.6)	56 (26.2)	6 (31.6)	0.595^1^
Liver, *n* (%)	68 (29.2)	63 (29.4)	5 (26.3)	1.000^1^
Spleen, *n* (%)	69 (29.6)	61 (28.5)	8 (42.1)	0.293^1^
Abdominal contamination, *n* (%)				0.167^1^
No hollow viscus injured	111 (47.6)	106 (49.5)	5 (26.3)	
Perforation. No visible contamination	36 (15.5)	32 (15.0)	4 (21.1)	
Contamination of a quadrant	39 (16.8)	34 (16.0)	5 (26.3)	
More than one quadrant contamination	46 (19.8)	41 (19.2)	5 (26.3)	
Location of the diaphragmatic wound				0.522^1^
Left, *n* (%)	151 (65.1)	139 (65.2)	12 (63.2)	
Right, *n* (%)	69 (29.7)	64 (30.1)	5 (26.3)	
Bilateral, *n* (%)	12 (5.2)	10 (4.7)	2 (10.5)	
AAST severity of the diaphragmatic trauma				0.535^1^
< 2	26 (11.2)	25 (11.7)	1 (5.3)	
2	179 (76.8)	162 (75.7)	17 (89.4)	
> 2	28 (12.0)	27 (12.6)	1 (5.3)	
Laparoscopic treatment	23 (9.9)	23 (10.8)	0 (–)	0.230^1^
Thoracic procedure				0.026^1^
Chest tube	144 (61.8)	137 (64.0)	7 (36.8)	
Transdiaphragmatic lavage	52 (22.3)	42 (19.6)	10 (52.6)	
Thoracotomy or thoracoscopy	29 (12.4)	28 (13.2)	1 (5.3)	
Chest tube + irrigation	8 (3.4)	7 (3.3)	1 (5.3)	

AIS, abbreviated injury scale; ATI, abdominal trauma index; AAST, The American Association for the Surgery of Trauma

^1^Fisher’s exact test

^2^Chi^2^

### Risk factors for empyema identification.

Table 3 reports the results of the simple logistic regression which identified as potential risk factors gunshot wounds, physiologic variables such as Glasgow Coma Scale and respiratory rate, trauma severity (measured by RTS, thoracic AIS > 3, ISS, NISS, ATI), associated trauma (facial trauma, hollow viscus injury, injuries of the stomach, colon, liver, spleen, and pancreas) and visible contamination. The severity of the diaphragmatic injury did not seem to affect the risk of empyema.

**Table 3:** Risk factors for empyema after diaphragmatic trauma. Logistic regression analysis

Variable	Univariate analysis		Multivariate analysis	
	O.R (95% C.I.)	*p*	O.R (95% C.I.)	*p*
Sex (female)	0.851 (0.186–3.895)	0.835		
Age, years	0.978 (0.930–1.028)	0.376		
Gunshot wound	3.389 (0.760–15.111)	0.110		
GCS	0.889 (0.761–1.039)	0.140		
RR	0.927 (0.846–1.015)	0.101		
RTS	0.685 (0.460–1.022)	0.064		
Facial trauma	1.523 (1.085–2.139)	0.015		
Thorax AIS > 3	3.719 (1.396–9.912)	0.009	4.270 (1.554–11.735)	0.005
ISS	1.024 (0.987–1.063)	0.200		
NISS	1.046 (1.016–1.078)	0.003	1.030 (0.996–1.066)	0.081
ATI	1.027 (0.999–1.055)	0.055		
OIS severity of the diaphragmatic injury	1.037 (0.564–1.909)	0.907		
Hollow viscus injury	1.052 (0.998–1.109)	0.061		
Stomach injury	2.492 (0.967–6.418)	0.059		
Colon injury	1.302 (0.472–3.590)	0.610		
Liver injury	0.856 (0.296–2.477)	0.774		
Spleen injury	1.824 (0.699–4.754)	0.219		
Pancreas injury	3.665 (1.191–11.285)	0.024		
Visible contamination	2.880 (1.002–8.278)	0.050	3.338 (1.127–9.888)	0.030
Transdiaphragmatic lavage	4.550 (1.739–11.904)	0.002		

GCS, Glasgow coma scale; RR, respiratory rate; RTS, revised trauma score; AIS, abbreviated injury scale; ISS, injury severity score; NISS, new injury severity score; ATI, abdominal trauma index; OIS, organ injury scale

**Fig. 1:**
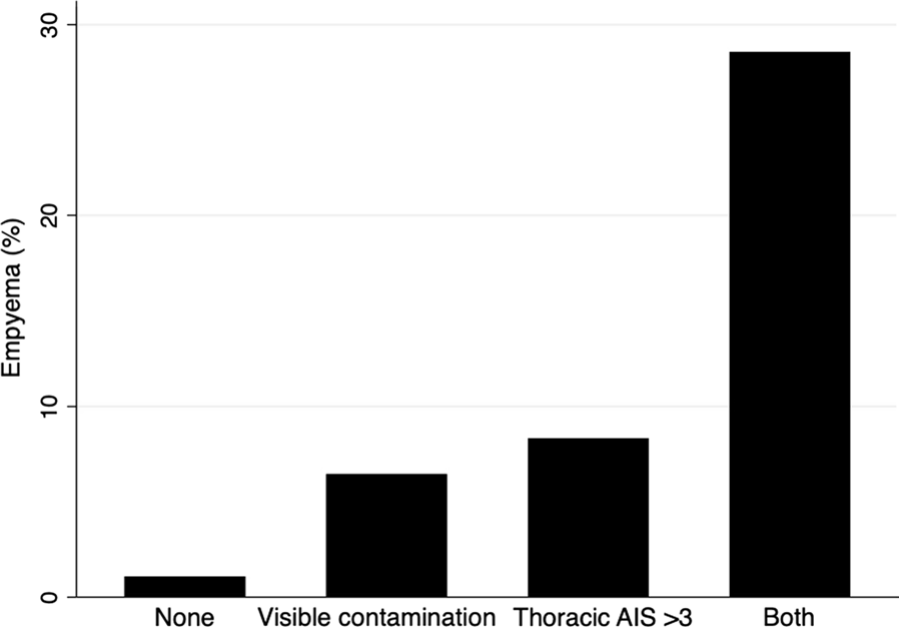
Incidence of empyema according to risk factors

After a selection process which aimed to identify the most representative variables such as the traumatic physiologic derangement on admission, the trauma severity, the associated injuries, and the contamination, the multiple logistic regression model retained as independent predictors the following variables: a severe thoracic trauma with an AIS > 3, O.R. (95% C.I.) 4.270 (1.554–11.735) and the presence of visible contamination, O.R. (96% C.I.) 3.338 (1.127–9.888) (Table 3). Figure 1 shows the increase of the empyema risk, regarding the presence of none, one or both identified risk factors. The model showed a good discriminative ability with an AUC of 0.7284 and a goodness of fit in the test of H–L.

**Fig. 2:**
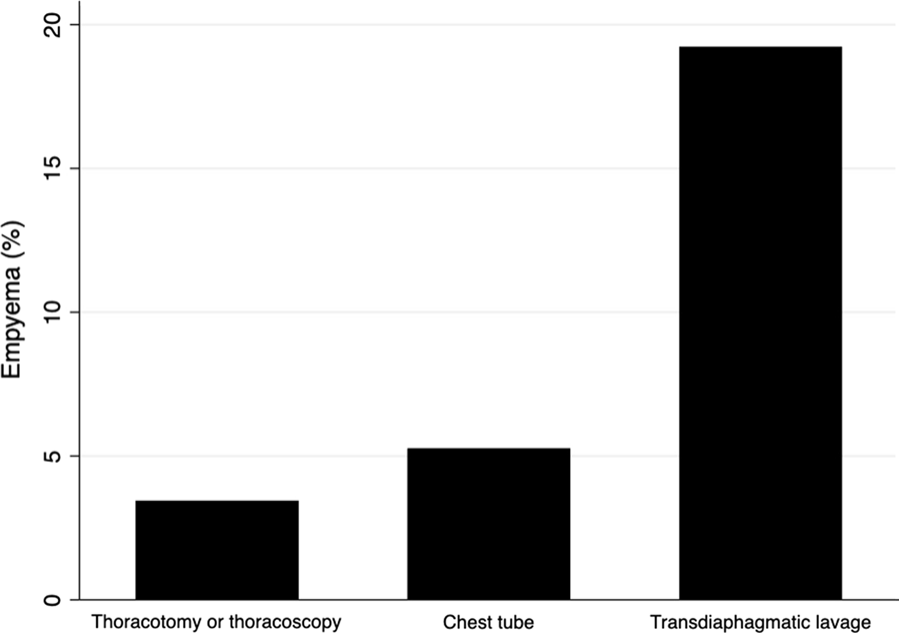
Contamination management and risk of empyema

The possible contribution of the procedures used to manage the pleural contamination is depicted in Fig. 2. Thoracoscopy or thoracotomy was associated with the lowest risk of empyema (3.4%), followed by chest tube (5.3%), and by TDPL, which showed the higher risk (19.2%) (Table 4). The association between TDPL and higher risk of empyema persisted after adjusting the variable by the risk factors identified, with an O.R. (95% C.I.) 10.589 (1.154–97.199) (Table 5).

**Table 4:** Risk factors for empyema after diaphragmatic trauma. Procedures used for the management of the thoracic contamination

Procedure	Number	Empyema *n* (%)
Chest tube	152	8 (5.3)
Thoracotomy or thoracoscopy	29	1 (3.4)
Transdiaphragmatic lavage	52	10 (19.2)*
Total	233	19 (8.2)

*p = 0.007 Fisher’s exact test

**Table 5:** Risk factors for empyema after diaphragmatic trauma. Relative contribution of the procedures used for the management of thoracic contamination

	O.R. (95% C.I.)	*p*
Unadjusted analysis		
Thoracotomy or thoracoscopy (reference)	1	–
Chest tube	1.556 (0.187–12.932)	0.683
Transdiaphragmatic lavage	6.667 (0. 808–55.016)	0.078
Adjusted analysis*		
Thoracotomy or thoracoscopy (reference)	1	
Chest tube	2.654 (0.291 24.229)	0.387
Transdiaphragmatic lavage	10.589 (1.154–97.199)	0.037

*Adjusted for thoracic AIS > 3 and visible contamination

## Discussion

The main purpose for this study was the need to clarify the risk factors for posttraumatic empyema in patients with penetrating diaphragmatic injuries. There is little information reported in literature regarding patients with penetrating diaphragmatic injuries and empyema. The incidence of posttraumatic empyema in patients with chest trauma ranges between 1.6 and 25% [11–15], representing one of the complications associated with chest tube placement and abdominal crossed-contamination in penetrating thoracoabdominal trauma [9, 10] and blunt trauma [17–20]. In this study, an incidence of posttraumatic empyema in patients with penetrating diaphragmatic injuries of 8.2% was reported. Similar to the ones presented by Karmy-Jones et al., who reported an incidence of 9% of posttraumatic empyema [21].

Meanwhile, little is known about the risk factors for posttraumatic empyema in patients with diaphragmatic injuries, the risk factors for empyema in patients with thoracic trauma and chest tube placement have been described [9, 10]. Aguilar et al. performed a retrospective review in 584 patients with chest tube due to thoracic trauma [10]. They reported an incidence of 4% of posttraumatic empyema [10]. A multiple logistic regression identified persistent pleural drainage (OR (95% CI) 12.5 (0.96–163)), pulmonary contusion (OR (95% CI) 6.3 (1.53–25.8)) and multiple chest tube insertion in the same hemithorax (OR (95% CI) 2.5 (1.91–3.28)) as independent factors of posttraumatic empyema [10]. Eren et al., in an analysis of risk factors of posttraumatic empyema in patients with thoracic injuries identified pulmonary contusion in patients with chest tube with an OR (95% CI) of 3.065 (1.744–5.386), retained hemothorax with an OR (95% CI) of 5.553 (3.25–9.48), laparotomy with an OR (95% CI) of 2.469 (1.512–4.032), chest tube duration > 6 days OR (95% CI) of 2.492 (1.526–4.042) and ICU length of stay > 2 days with an OR (95% CI) of 4.211 (2.511–7.062) [9]. Our study identified a thoracic AIS > 3 in the multivariate analysis, as a risk factor of posttraumatic empyema.

Several scientific articles report that contamination of the pleural cavity with bilio-gastroenteric contents represents an increase in the risk of posttraumatic empyema in patients with diaphragmatic injuries [1, 2, 15, 17, 18]. Coimbra et al. performed an analysis in patients with gastric injuries in the development of post-operatory complications [18]. They described that post-operatory pleuropulmonary complications were more frequent in patients with diaphragmatic injuries compared to patients without diaphragmatic injuries (26.9% vs 8.5%). And that 62.5% of the complications were present in the gross contamination group [18]. Nevertheless, in this report, only bivariate associations were performed. The lack of multivariate analysis weakens the reported findings. Meanwhile, in our study the univariate analysis showed a tendency of gastric injury to increase the risk of posttraumatic empyema with an OR (95% CI) of 2.492 (0.967–6.418), which was not confirmed in the multivariate analysis. Instead, the multivariate analysis identified visible contamination as an independent risk factor explaining the tendency of gastric injury observed in the univariate analysis, extending the exposure to other hollow viscus injuries.

Barmparas et al. performed a retrospective analysis using the National Trauma Databank of risk factors for empyema after diaphragmatic injuries [2]. They separated patients in 2 groups depending on the presentation of posttraumatic empyema. The stepwise logistic regression identified gastric injury (OR(95% CI) 2.9 (1.69–5)) and ISS > 20 (OR(95% CI) 2.99 (1.61–5.59)) as independent risk factors for posttraumatic empyema [2]. They proposed TDPL and chest tube placement in index surgery as a preventive strategy for posttraumatic empyema but did not provide data to support the recommendation [2].

In our study, when comparing the incidence of empyema according to each risk factor (graph 1), some situations must be considered. As seen in the graph when neither thoracic AIS > 3 nor visible contamination was present, there was an approximate incidence of 1% of posttraumatic empyema. This suggests that in cases were none of the identified risk factors are present, pleural cavity lavage or other additional methods to mitigate the pleural contamination might not represent an additional benefit to prevent posttraumatic empyema. It also means that in a low incidence there will be cases of empyema despite having no risk factors. In addition, when having both risk factors, thoracic AIS > 3 and visible contamination, empyema incidence rose significantly to approximately 28%, which indicating that this group of patients might benefit from therapeutic strategies such as video-assisted thoracoscopy.

Some investigators have proposed strategies as TDPL [1, 2]. Zellweger et al. performed a retrospective review of TDPL in a selected group of patients with thoracoabdominal trauma and diaphragmatic injury as a method to prevent posttraumatic empyema [1]. They reported 20% of complications and 6% of complications in the pleural cavity, among them, 2 cases of empyema (1%) [1]. Unfortunately, the methodology used in this study with no control group does not provide enough evidence to recommend this strategy as a preventive mechanism or to establish a causal nature in the development of posttraumatic empyema. Additionally, the severity of the trauma reported with a median (IQR) ISS of 38.1 (35–50) and no data regarding mortality [1] is not consistent with our results or with those reported in the literature [22, 23].

**Fig. 3:**
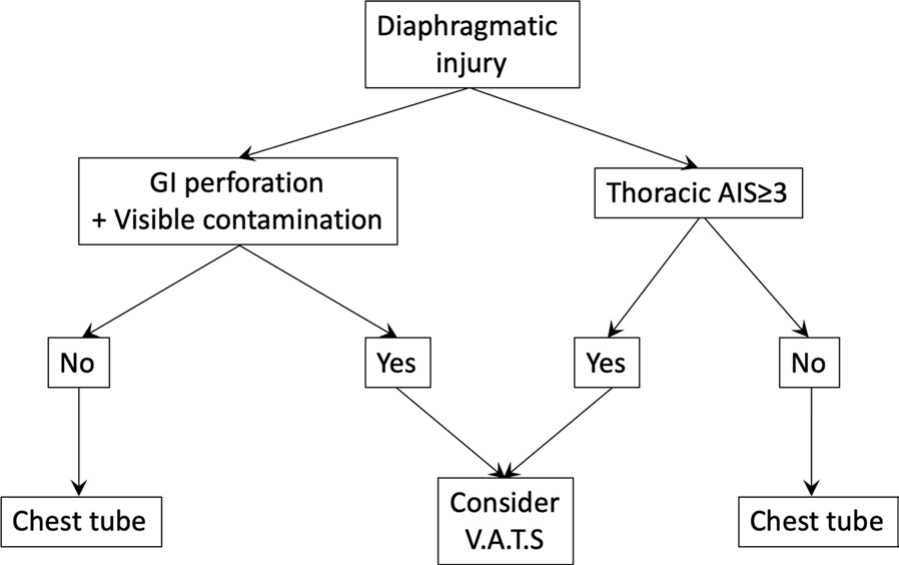
Algorithm for empyema prevention in diaphragmatic penetrating injury

Interestingly, in our study, the analysis of the contribution to the risk of empyema of the procedures used in the index surgery for the management of thoracic contamination showed that TDPL exhibited a significant association with higher risk, even after adjusting for the identified risk factors: thoracic AIS > 3 and visible contamination. Our results do not agree with the only report available to date in which this technique has been reported. The technique used by our surgeons and the criteria for stopping the lavage are similar to the work published by Zellweger et al. [1]. Our report seems more robust, as we compared the TDPL with other procedures and adjusted the result for the trauma severity and the contamination, thus better controlling the possible confounding effect related to these variables. In Fig. 3, we present an algorithm in the management of diaphragmatic penetrating injury, considering the results of our analysis.

### Limitations

Our study has several limitations. First, its retrospective nature gives the risk of information bias. Second, the small number of patients with the studied outcome limits the multivariate analysis. Nevertheless, the candidate risk factor variables were progressively selected, reaching a robust model. And third, we did not include individual variables regarding specific thoracic injuries, such as rib fractures, pulmonary lacerations and pulmonary contusions. This decision was taken, given that most of the patients in our cohort were neither operated on nor studied with CT scan. Therefore, a specific determination of the characteristics of pulmonary injuries could not be included in a vast majority of the cases. Additionally, the AIS scoring where the most severe injury prevails over the others was mainly influenced by the magnitude of the hemothorax.

### Strengths

The multivariate analysis permitted us to deal with confounding and explore the role of trauma severity, injured organs, contamination, and surgical procedures. The charts review led to surgical details not explored in some of the previous studies.

## Conclusion

In patients with penetrating diaphragmatic injuries, risk factors for posttraumatic empyema such as thoracic AIS > 3 and visible contamination must be considered. Strategies for managing thoracic and abdominal cavity contamination are justified when both risk factors are present. Nevertheless, considering our results when TDPL was performed, this technique must be used cautiously until a randomized control clinical trial is conducted to assess its effectiveness in preventing posttraumatic empyema in this subset of patients.

## Data Availability

Due to the nature of the study and in favor of protecting the identity of each patient, the institutional review board did not agree for the data to be shared, so supporting data and materials are not available. Methods are described in the respective section in the manuscript.
